# “Physiological effects of high-intensity versus low-intensity noninvasive positive pressure ventilation in patients with acute exacerbation of chronic obstructive pulmonary disease: a randomised controlled trial”

**DOI:** 10.1186/s13613-022-01044-2

**Published:** 2022-07-11

**Authors:** Radhouane Toumi, Khaoula Meddeb, Mohamed Boussarsar

**Affiliations:** 1 Medical Intensive Care Unit, Farhat Hached University Hospital, Sousse, Tunisia; 2 Research Laboratory Heart Failure, LR12SP09. Farhat, Hached University Hospital, Sousse, Tunisia; 3 Faculty of Medicine of Sousse, University of Sousse, Sousse, Tunisia

Dear editor

We read with great interest the article of Luo et al. “Physiological effects of high-intensity versus low-intensity noninvasive positive pressure ventilation in patients with acute exacerbation of chronic obstructive pulmonary disease: a randomised controlled trial” published in *Annals of Intensive Care* [1]. In this physiological trial, the authors hypothesized that noninvasive positive pressure ventilation (NPPV) using higher levels of pressure would be superior to using lower levels of pressure in reducing elevated arterial carbon dioxide tension (PaCO_2_), inspiratory effort, improving consciousness and NPPV tolerance in patients with acute exacerbation of chronic obstructive pulmonary disease (AECOPD). While this trial provided positive results encouraging further trials on this subject, a few reservations could be raised.

First, as stated in the title of the manuscript, this study focuses on the physiological effects of the intervention. We notice however, that the authors overlooked an important physiological parameter which is the pH level. The mean pH level significantly differs as of 24 h of NPPV. The pH tends to become alkaline in the high-intensity NPPV group while it remains neutral in the low-intensity NPPV. It is important to point out that the increase in pH levels exposes patients to compensatory hypoventilation, which has been well established in the past [2] and to hyperventilation-triggered seizures in which alkalosis has been suggested as a cause [3]. An important reminder is that decreasing the levels of PaCO2 is only a bridge to recovery as the main biological criteria for NPPV success while treating AECOPD is obtaining a pH > 7.35 [4]. This is a factor that should be discussed seen as, according to these results, high-intensity NPPV could expose patients to both hypoventilation and seizures.

Second, while we value that the authors reported ventilatory settings and physiological parameters in the supplementary material, we believe more data should have been provided in order to better characterize patients’ respiratory mechanics for a more thorough comparability and generalizability. Regarding NPPV settings and for a more comprehensive and physiological approach, we should keep in mind that the tidal volume generated while on NPPV does not solely depend on the pressure support level. In fact, beyond the patient’s effort and pressure support, the inspiratory pressurization slope and expiratory trigger sensitivity also contribute to generating the tidal volume. The authors failed to address these parameters. In order to demonstrate that the levels of pressure support along with the daily hours of NPPV were the only factors significantly different between both groups, it would have been subtler to show the comparability of these patients in terms of inspiratory slope and expiratory trigger sensitivity usually adjusted to offset leaks and prevent patient–ventilator interaction by adjusting the mechanical drive to the neural drive.

As for NPPV monitoring, while we appreciate that the authors excluded emphysematous patients, we think it would have been very elegant to appreciate the degree of pulmonary distention in both groups by analyzing intrinsic positive end-expiratory pressure (PEEP) and both slopes of the expiratory flow curve. Intrinsic PEEP can be estimated by noting the first values of pressure displayed while performing an end-expiratory occlusion in volume control mode with a high inspiratory trigger. Visually analyzing expiratory flow curves can also estimate the degree of obstruction as well as gas trapping.

Finally, we notice that the NPPV in this study was performed using a hybrid ventilator with a single-limb circuit with intentional air leaks, which is indirectly concluded by the high leakage values displayed in the supplementary material as well as by the type of ventilator used. We wonder to which extent these results are reproducible and generalizable to patients undergoing NPPV in intensive care units using double-limb circuits with a demand valve and an expiratory valve.

Using high-intensity NPPV in AECOPD is interesting to further evaluate in clinical randomized controlled trials. After addressing the question of generalizability to all ventilators, we suggest including additional ventilatory settings and monitoring parameters as well as assessing risks of rapid-induced post-hypercapnic alkalosis in upcoming studies.

## Data Availability

None.

## Abbreviations

NPPV: Noninvasive positive pressure ventilation
PaCO2: Arterial carbon dioxide tension
AECOPD: Acute exacerbation of chronic obstructive pulmonary disease
PEEP: Positive end-expiratory pressure
